# Intermittent exogenous ketosis increases erythropoietin in healthy adults: A randomized, placebo‐controlled study with preliminary sex‐specific observations

**DOI:** 10.14814/phy2.71041

**Published:** 2026-08-03

**Authors:** Henrik Holm Thomsen, Birgitte Sandfeld‐Paulsen, Lise Nørkjær Bjerg, Jesper Nørgaard Bech

**Affiliations:** ^1^ Medical Diagnostic Center University Clinic for Innovative Patient Pathways, Regional Hospital Central Jutland Viborg Denmark; ^2^ Department of Clinical Medicine Aarhus University Aarhus Denmark; ^3^ Department of Clinical Biochemistry Regional Hospital Central Jutland Viborg Denmark; ^4^ Department of Clinical Biochemistry Aalborg University Hospital Aalborg Denmark; ^5^ University Clinic in Nephrology and Hypertension Gødstrup Regional Hospital Herning Denmark

**Keywords:** anemia, erythropoietin, ferritin, iron, ketone ester, ketosis

## Abstract

Exogenous ketosis may stimulate erythropoietin (EPO), yet its impact on erythropoiesis, iron metabolism, and potential sex‐specific responses in humans remains unknown. We investigated these effects in healthy adults using oral β‐hydroxybutyrate ketone monoester (KE) to induce intermittent ketosis. Acute EPO responses to a single dose of KE were assessed in 16 healthy adults (pre‐post), followed by a 2‐week randomized study (KE twice daily vs. placebo). After 2 weeks, EPO levels with intermittent ketosis were approximately 30% higher (95% CI 6, 60), *p* = 0.01, an effect more evident in females (9.7 IU/L (95% CI 6.0, 13.5) vs. 6.4 IU/L (95% CI 4.0, 8.8), *p* = 0.008) than in males (10.7 IU/L (7.2, 14.2) vs. 9.6 IU/L (6.3, 12.9), *p* = 0.46). Ferritin was elevated in females with KE vs. placebo (42 μg/L vs. 16 μg/L, *p* < 0.001). Hemoglobin and red blood cell count were unaffected at 2 weeks. Over 2 weeks, intermittent exogenous ketosis was associated with a modest increase in erythropoietin levels, with a stronger signal in females. Although hemoglobin and red blood cell count did not change significantly, females showed higher ferritin levels and a trend toward increased hematocrit, suggesting subtle effects on erythropoietic stimulation and iron handling.

## Graphical abstract

Intermittent exogenous ketosis stimulates erythropoietin, with greater EPO response and elevated ferritin in females but no significant changes in circulating hemoglobin or red blood cell count at 2 weeks.

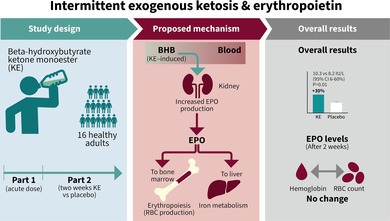

## 1 INTRODUCTION

Erythropoietin (EPO) is a pivotal hormone that regulates erythropoiesis by stimulating the proliferation and maturation of erythroid progenitor cells in the bone marrow. Its synthesis is primarily governed by oxygen availability. Reduced renal oxygen supply secondary to low hematocrit or blood volume triggers EPO production as a compensatory mechanism to restore oxygen delivery. Beyond hypoxia, multiple non‐hypoxic pathways influence EPO regulation (Jelkmann, 2011), including various hormones, particularly sex hormones (Bachman et al., 2014; Suresh et al., 2019), and nutritional components. Cytokines can have various functions, with pro‐inflammatory cytokines such as interleukin‐1β and tumor necrosis factor‐α potentially suppressing EPO production, while others may have a stimulating effect (Cluzeau et al., 2017; Wu et al., 2025). Considering EPO's central role in maintaining red blood cell mass, the regulation of EPO by hormonal and pharmacological factors remains an important area of investigation.

Experimental research exploring exogenous ketosis has provided preliminary support for a direct stimulatory effect on EPO. Administration of racemic sodium BHB has been shown to elevate circulating EPO levels and increase bone marrow (Howard et al., 2024) F‐fluorodeoxyglucose uptake (Lauritsen et al., 2018). Similarly, athletes consuming a ketone monoester drink exhibited transient increases in EPO levels, consistent with augmented EPO signaling (Evans et al., 2023). Other endurance training studies have reported comparable findings, but they did not demonstrate a clear correlation between blood BHB and EPO levels (Poffé et al., 2023). Collectively, these data suggest that exogenous ketosis may stimulate EPO production, although a consistent link to downstream erythropoietic responses has not been established. Specifically, the extent to which ketosis modulates EPO, erythropoiesis, and iron metabolism in humans, and whether these effects differ between sexes, has not been systematically investigated. Experimental work in rodents indicates possible sex‐specific responses to EPO, with estrogen implicated in modifying metabolic outcomes, which may influence EPO regulation (Zhang et al., 2017). However, similar investigations in humans are limited and inconclusive (Yin & Noguchi, 2025).

Metabolic interventions that alter substrate availability provide further support for such regulation. Inhibiting renal glucose reabsorption with SGLT2i increases both renal EPO expression and circulating EPO concentrations, and even modest elevations in circulating BHB appear to enhance EPO levels (Kanta et al., 2025). Research on the mechanistic connection between ketosis and erythropoiesis is limited, and the physiological significance of this association during exogenous ketosis has not been adequately investigated.

We hypothesized that intermittent exogenous ketosis would increase erythropoietin levels and enhance erythropoiesis. Therefore, we conducted a randomized controlled trial in healthy adults. Both male and female participants were included to explore potential sex‐stratified responses in EPO and sex hormones, although sex‐specific effects were not primary objectives. Tolerability and adverse events were monitored systematically throughout the intervention. Secondary analyses evaluated acute metabolic responses to ketosis using a pre–post design without a control group.

## 2 MATERIALS AND METHODS

### 2.1 Study design

This study was conducted in two parts. Part 1 was a quasi‐experimental, one‐group, pre‐post design in which all participants ingested a BHB KE drink (Figure S1), serving as the acute effects study. Part 1 served as the data source for previously published methodological studies on BHB measurement agreement across sampling sites and by a continuous ketone monitoring device (Bjerg et al., 2025; Kjær et al., 2026). No erythropoietic or hematological data from the present study have been reported elsewhere. Part 2, the long‐term effects study, was a randomized controlled trial with a 1:1 allocation to BHB KE or placebo (PBO). After all participants had been enrolled and assigned sequential study identification numbers in order of enrolment, a simple random allocation list (no stratification or blocking) was generated using Randomizer.org, linking each identification number to one of the two interventions. Participants and laboratory personnel were blinded to the intervention, but the investigators were not.

### 2.2 Statistical analyses

Data from Parts 1 and 2 were analyzed using linear mixed‐effects models with a single combined categorical factor representing each unique time × treatment combination. Pre‐randomization levels were shared across all 16 participants (common baseline anchor), while day 14 had separate levels for the KE and PBO arms, allowing acute pre–post (Part 1) and randomized comparisons (Part 2) within a single model. Participant was included as a random effect, and the within‐participant correlation over time was modeled through an unstructured residual covariance by time. Age, sex, and menstrual status were not used as stratification factors and were not considered during randomization.

Two fixed‐effects structures were used. For hematological and iron outcomes, the structure comprised the full factorial of time × treatment by sex. For sex hormones, cell‐means parametrisation was used (no intercept) to accommodate substantial between‐sex differences in baseline concentrations and variability. Log‐transformed data are reported as geometric means with 95% confidence intervals (CI); other outcomes as estimated marginal means with 95% CIs.

Exploratory associations between EPO and sex hormones were examined using linear regression with cluster‐robust standard errors on log‐transformed values, stratified by timepoint (baseline and day 14) and sex, to account for repeated measurements within individuals. Sex‐specific effects are presented as stratified within‐sex contrasts rather than formal interaction tests, given limited power for effect modification. Pairwise comparisons were performed using effect‐coded contrasts without multiplicity adjustment, consistent with the exploratory nature of secondary analyses. Model assumptions were assessed through residual diagnostics. Statistical significance was set at *p* < 0.05. *p*‐values less than 0.001 are reported as *p* < 0.001. Analyses were performed using Stata 18 (StataCorp LLC, College Station, TX, USA).

#### 2.2.1 Sample size calculations

The sample size calculation was based on the primary outcome of EPO concentration after 2 weeks of intervention (Part 2). Using previously reported mean EPO concentrations of 9.96 IU/L (SD 1.1) and 7.6 IU/L (SD 1.0) (Lauritsen et al., 2018), a sample size of five participants was required, given an alpha of 0.05 and a beta of 10%. Modeling with a 50% reduced mean difference and 0.5 correlation yielded *N* = 11. We increased the sample size to 16 participants in total (eight in each group in Part 2) to enhance the statistical power of the study and mitigate the impact of potential data attrition. This increase in n was also critical for securing a more robust number of measurements in Part 1. Although this was a secondary aim, including both male and female participants with this larger sample size facilitated the exploration of potential sex‐based inferences concerning the studied mechanisms.

### 2.3 Participants

Participants were recruited through hospital board announcements. The inclusion criteria were age 18–60 years and body mass index of 19–30 kg/m^2^. The study excluded those unable to comprehend or provide consent or fulfill the study requirements. Individuals with electrolyte disorders, kidney disease, or liver or bile disease were excluded from participation. Additional exclusion criteria were diabetes mellitus, metabolic and hormonal diseases, use of illegal medications, anemia or hematopoietic diseases, previous bariatric surgery, previous myocardial infarction or uncontrolled myocardial ischaemia, recent weight loss, and allergies to catheters or adhesives. The study was approved by the Central Denmark Region Committee on Biomedical Research Ethics (1‐10‐72‐221‐22), registered at ClinicalTrials.gov (NCT06053138), and conducted in accordance with the Declaration of Helsinki. Oral and written informed consent was obtained from all participants.

### 2.4 Intervention

In Part 1, all participants ingested a BHB ketone monoester (KE) beverage (KE4, KetoneAid Inc., Falls Church, VA, USA) at 500 mg/kg, administered in two portions: two‐thirds initially and one‐third after 60 min. In Part 2, participants received daily doses of either 500 mg/kg BHB KE or a PBO beverage for 2 weeks. The PBO beverage contained no ketone ester and provided no caloric value. It was prepared in‐house by the investigators according to a placebo formulation supplied by the manufacturer of the ketone ester (KetoneAid Inc., Falls Church, VA, USA) and was matched to the active drink for volume, taste, and viscosity. The PBO beverage comprised distilled water thickened with arrowroot, stevia, a bittering agent (denatonium benzoate), and potassium sorbate as preservative, without the optional cherry flavoring, thereby matching the taste profile of KE4. Both interventions were dispensed in neutral, identical, opaque bottles. Fifty percent of the daily dose was administered at bedtime, and the remainder was provided in one or two installments, with a minimum six‐hour interval, according to participant preference, thereby establishing intermittent ketosis. Administering 50% of each daily dose at bedtime was intended to prolong the overnight, fasted window of supraphysiological ketosis, because concurrent food or carbohydrate intake lowers the circulating BHB excursion after exogenous ketone ingestion, primarily through insulin‐mediated enhancement of BHB clearance and oxidation rather than suppression of hepatic ketogenesis (Stubbs et al., 2017). At the two‐week assessment, blood was sampled in the morning after an overnight fast from food. The final KE or PBO dose had been taken at bedtime the preceding evening, in keeping with the intermittent dosing schedule.

### 2.5 Analyses

Blood samples were collected in the Department of Clinical Biochemistry, Regional Hospital Central Jutland, Viborg. Venous samples for testosterone, oestradiol, BHB, EPO, and sex hormone‐binding globulin (SHBG) were allowed to coagulate for 1 h before centrifugation at 3000*g* for 5 min. The serum was stored at −80°C until analysis. Testosterone, oestradiol, and BHB levels were measured using high‐performance liquid chromatography tandem mass spectrometry (HPLC‐MS/MS) (Sørensen et al., 2013). EPO was measured using an IMMULITE 2000 analyzer (Siemens Healthineers AG, Forchheim, Germany) with an associated chemiluminescent immunoassay (IMMULITE 2000 EPO REF: L2KEPN2), while SHBG was measured using an electrochemiluminescence immunoassay reagent (Elecsys SHBG REF. 03052001) on the Cobas E801 (Roche Diagnostics, Rotkreuz, Switzerland). Iron, ferritin, transferrin, luteinizing hormone, and follicle stimulating hormone levels were quantified in plasma using the Atellica CH 930 modules and reagents (Atellica CH Iron_2 REF. 11097601; Atellica CH Transferrin REF. 11097597) as well as Atellica IM modules with related reagents (Atellica IM Ferritin REF: 10995568; Atellica IM Luteinizing Hormone REF. 10995635; Atellica IM Follicle Stimulating Hormone REF: 10995580) (Siemens Healthineers AG, Forchheim, Germany). The Sysmex XN‐9100 (Sysmex Corporation, Kobe, Japan) was used to analyze and calculate hematological parameters in whole blood.

### 2.6 Harms

Adverse events were assessed at each study visit using open‐ended questioning, asking participants to report any symptoms or tolerability issues they had experienced.

## 3 RESULTS

### 3.1 Participants

Sixteen participants (9 males) completed the study (Figure S2) Part 1 and continued to Part 2. They had a median age of 42 (range 31–54) years, and a median body mass index (BMI) of 24.2 kg/m^2^ (range 19.8–30.7). No adverse events were reported in either group. Both the KE and PBO interventions were rated as having poor palatability by most participants, with no difference between groups in the frequency or intensity of taste complaints. No other tolerability issues were identified. All nine male participants in the study showed normal testicular function on clinical and biochemical assessment. Among the female participants, one was undergoing hormone replacement therapy, and one was perimenopausal. Of the remaining five, three were in the follicular phase and two were in the luteal phase, both using a progestin‐releasing intrauterine device; phases were assigned based on menstrual cycle history, follicle‐stimulating hormone, luteinizing hormone, and oestradiol levels. Consequently, some female participants were tested during different cycle phases in Parts 1 and 2.

### 3.2 Part 1: Acute effects of BHB ketone monoester ingestion

#### 3.2.1 β‐Hydroxybutyrate

Plasma BHB levels rose rapidly following BHB KE ingestion, peaking at 120 min at 3.5 mmol/L (3.3, 3.8) versus a pre‐ingestion concentration of 0.04 mmol/L (0.03, 0.05), *p* < 0.001, Table 1.

**TABLE 1:** Part 1 – Acute effects of ketone monoester ingestion.

	Baseline	180 min	*p*‐value
BHB, mmol/L^a^	0.04 (0.03, 0.05)	2.4 (2.1, 2.7)	<0.001
EPO, IU/L^a^	8.1 (6.3, 10.0)	8.3 (6.5, 10.1)	0.73
Hematocrit, %	40 (38, 41)	40 (38, 41)	0.50
Hemoglobin, mmol/L	8.5 (8.1, 8.8)	8.4 (8.1, 8.8)	0.99
RBC, ×10^12^/L	4.5 (4.3, 4.7)	4.5 (4.3, 4.7)	0.97
MCHC, mmol/L	21.4 (21.1, 21.6)	21.3 (21.1, 21.5)	0.43
MCV, fL	88 (86, 90)	89 (87, 91)	0.04
Plasma iron, μmol/L	15.2 (12.2, 18.2)	21.5 (18.2, 24.7)	<0.001
TSAT, %	30 (24, 35)	41 (35, 48)	<0.001
Ferritin, μg/L^a^	75 (48, 102)	75 (48, 103)	0.71

*Note*: Acute effects of a single BHB ketone monoester dose (part 1, one‐group pre‐post design; *N* = 16). Data are means (95% CI) at baseline and 180 min from the linear mixed‐effects model.

Abbreviations: BHB, β‐hydroxybutyrate; EPO, erythropoietin; MCHC, mean corpuscular hemoglobin concentration; MCV, mean corpuscular volume; RBC, red blood cell count; TSAT, transferrin saturation.

^a^ Log‐transformed variable; values are geometric means (95% CI).

#### 3.2.2 Erythropoietin and erythrocyte parameters

EPO concentrations were not affected by acute ketosis (baseline 8.1 IU/L (6.3, 10.0) vs. 8.3 IU/L (6.5, 10.1) at 180 min, *p* = 0.73; Table 1). Hemoglobin was also stable across the trial day (difference at 180 min vs. baseline: 0.0 mmol/L, *p* = 0.99). Hematocrit increased modestly at 90 min relative to baseline by 0.7 percentage points (0.3, 1.0), *p* < 0.001, but returned to baseline by the end of the trial day, *p* = 0.50. Red blood cell count (RBC) remained unchanged (*p* = 0.97). A slight increase in MCV was observed at 180 min versus baseline (difference 0.4 fL (0.02, 0.7), *p* = 0.04).

#### 3.2.3 Iron status

Plasma iron increased sharply from 15.2 μmol/L (12.2, 18.2) at baseline to 21.5 μmol/L (18.2, 24.7) at 180 min, *p* < 0.001. Transferrin saturation (TSAT) increased concomitantly by 12.0 percentage points (8.8, 15.2) at 180 min, *p* < 0.001. No acute effect on ferritin was observed in either sex. The acute increases in plasma iron and TSAT were not significantly different between males and females.

### 3.3 Part 2: Effects of two‐week intermittent ketosis with BHB ketone monoester

#### 3.3.1 β‐Hydroxybutyrate

At the two‐week assessment, BHB concentrations were 0.9 mmol/L (0.1, 1.6) with KE versus 0.02 mmol/L (0.005, 0.04) with PBO, *p* < 0.001, confirming intermittent ketosis throughout the intervention.

#### 3.3.2 Erythropoietin

**FIGURE 1:**
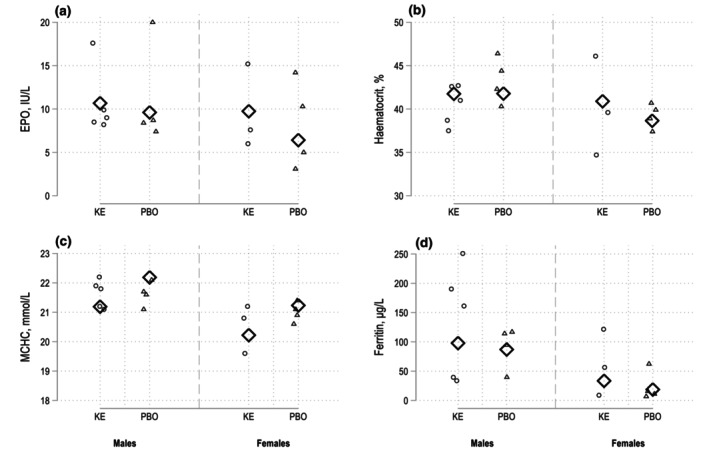
Hematological and iron markers. Erythropoietin (EPO) (a), hematocrit (b), mean corpuscular hemoglobin concentration (MCHC) (c), and ferritin (d) after 2 weeks of intermittent ketosis (KE) or placebo (PBO). Individual observations (circles for KE, triangles for PBO) and model‐derived marginal means (hollow diamonds) from linear mixed‐effects models are shown. Males (left panels) and females (right panels) are displayed separately (*N* = 16; 9 males, 7 females).

After 2 weeks, EPO concentration was 30% (6, 60) higher with KE than with PBO: 10.3 IU/L (7.7, 12.8) versus 8.2 IU/L (6.1, 10.3), *p* = 0.01 (Table 2 and Figure 1a). The effect was more pronounced in females, in whom EPO was 52% (11, 107) higher with KE (9.7 IU/L (6.0, 13.5)) than with PBO (6.4 IU/L (4.0, 8.8)), *p* = 0.008. In males, the between‐group difference was 11% (−16, 47) and was not statistically significant, *p* = 0.46.

**TABLE 2:** Part 2 – Two weeks of intermittent exogenous ketosis vs. placebo.

	KE	PBO	*p*‐value
BHB, mmol/L	0.9 (0.1, 1.6)	0.02 (0.005, 0.04)	<0.001
EPO, IU/L^a^	10.3 (7.7, 12.8)	8.2 (6.1, 10.3)	0.01
Males	10.7 (7.2, 14.2)	9.6 (6.3, 12.9)	0.46
Females	9.7 (6.0, 13.5)	6.4 (4.0, 8.8)	0.008
Hematocrit, %	41 (40, 43)	40 (39, 42)	0.21
Males	42 (39, 44)	42 (39, 44)	0.98
Females	41 (38, 44)	39 (36, 41)	0.09
Hemoglobin, mmol/L	8.6 (8.2, 9.0)	8.8 (8.4, 9.2)	0.33
RBC, ×10^12^/L	4.6 (4.4, 4.8)	4.6 (4.4, 4.8)	0.79
MCHC, mmol/L	20.8 (20.4, 21.1)	21.8 (21.4, 22.1)	<0.001
Males	21.2 (20.7, 21.7)	22.2 (21.7, 22.7)	<0.001
Females	20.2 (19.7, 20.8)	21.2 (20.7, 21.8)	<0.001
MCV, fL	89 (87, 91)	89 (87, 91)	0.45
Plasma iron, μmol/L	16.6 (12.8, 20.4)	17.6 (13.9, 21.4)	0.74
TSAT, %	32 (24, 40)	32 (24, 40)	0.96
Ferritin, μg/L^a^	72 (42, 102)	57 (32, 83)	<0.001
Males	96 (49, 143)	89 (45, 133)	0.46
Females	42 (9, 75)	16 (3, 28)	<0.001

*Note*: Effects of 2 weeks of intermittent exogenous ketosis on hematological and iron parameters (Part 2, randomized controlled trial; *N* = 16: KE *N* = 8, PBO *N* = 8). Data are means (95% CI) on Day 14 from the linear mixed‐effects model. *p*‐values refer to the model‐estimated KE versus PBO contrast on Day 14, equivalent to the baseline‐anchored time × intervention interaction. Sex‐stratified sub‐rows were exploratory.

Abbreviations: BHB, β‐hydroxybutyrate; EPO, erythropoietin; KE, ketone monoester; MCHC, mean corpuscular hemoglobin concentration; MCV, mean corpuscular volume; PBO, placebo; RBC, red blood cell count; TSAT, transferrin saturation.

^a^ Log‐transformed; values are geometric means.

#### 3.3.3 Erythrocyte parameters

Hemoglobin was unaffected by the intervention at 2 weeks: KE versus PBO difference 0.2 mmol/L (−0.2, 0.6), *p* = 0.33 (Figure 1b). In females, a trend toward a higher hematocrit with KE was observed (40.9% (38.1, 43.7) vs. 38.7% (36.0, 41.3), *p* = 0.09), whereas no difference was detected in males (Figure 1c). RBC was not significantly affected by the intervention (KE vs. PBO: *p* = 0.79). MCV was similarly unchanged at 2 weeks (KE vs. PBO difference 0.4 fL (−0.6, 1.4), *p* = 0.45). In contrast, mean corpuscular hemoglobin concentration (MCHC) was lower with KE than with PBO at 2 weeks (difference 1.0 mmol/L (0.7, 1.3), *p* < 0.001), and this reduction was consistent across sexes.

#### 3.3.4 Iron status

No significant between‐group differences in plasma iron or TSAT were observed at 2 weeks (*p* = 0.74 and *p* = 0.96, respectively). The overall group ferritin level was higher with KE (72 μg/L (42, 102)) than with PBO (57 μg/L (32, 83)), *p* < 0.001 (Table 2). This difference was confined to female participants, in whom ferritin levels were significantly higher with KE (42 μg/L (9, 75)) than with PBO (16 μg/L (3, 28)), *p* < 0.001 (Figure 1d). No such effects were observed in males.

#### 3.3.5 Sex hormones

Males had substantially higher testosterone than females (mean difference 17.4 nmol/L (14.1, 20.6), *p* < 0.001), and females had higher oestradiol levels than males (459 pmol/L (85, 834) vs. 89 pmol/L (72, 106), *p* = 0.001). In females, testosterone was 70% (52, 92) higher with KE than with PBO at 2 weeks (1.2 nmol/L (0.8, 1.6) vs. 0.7 nmol/L (0.5, 0.9), *p* < 0.001). No corresponding effects were detected in males. SHBG levels were not significantly affected by the intervention in either sex (*p* = 0.74).

In an exploratory analysis, EPO was inversely associated with both oestradiol and testosterone at baseline in females (oestradiol: *β* = −0.32 (−0.44, −0.20), *R* ^2^ = 0.62, *p* = 0.001; testosterone: *β* = −0.79 (−1.35, −0.22), *R* ^2^ = 0.50, *p* = 0.014). These associations were not observed at day 14 in females (oestradiol, *p* = 0.47; testosterone, *p* = 0.39) or in males at either timepoint (all *p* > 0.36). These correlations are presented in Figures S3 (testosterone) and S4 (oestradiol).

## 4 DISCUSSION

This randomized study shows that a two‐week protocol of intermittent exogenous ketosis from KE supplementation modestly increases EPO levels and enhances iron availability, especially in females.

Although females have lower EPO concentrations, in part due to reduced androgen stimulation and increased oestradiol inhibition (Suresh et al., 2019), ketosis can elicit EPO production in women. Following a two‐week period of intermittent ketosis, the EPO levels in females equalled those observed in males. Additionally, there was a noted trend of increased hematocrit in females, a pattern not observed in their male counterparts. Also, MCHC decreased in both sexes, indicating an early bone marrow response.

### 4.1 Erythropoietin

Some have reported a decrease in EPO levels with ketosis in patients with heart failure (Berg‐Hansen et al., 2024; Berg‐Hansen et al., 2025), which contrasts with our findings in younger healthy adults. Baseline physiological state and inflammatory burden may substantially influence the direction of the EPO response to exogenous ketosis. Our lack of acute EPO effects aligns with other oral BHB studies (Howard et al., 2024; Mosquera‐Lopez et al., 2026; Stalmans et al., 2025), suggesting that other factors might influence acute changes rather than direct erythropoietic signaling.

While the underlying pathways by which BHB may influence EPO remain unclear, several mechanistic hypotheses have been proposed, including hypoxia‐inducible factor stabilization (Semenza, 2023), inhibition of histone deacetylases (Shimazu et al., 2013), and a shift toward an increased NAD+/NADH ratio during nutritional ketosis (Xin et al., 2018). These mechanisms are speculative in the context of human exogenous ketosis, as we did not measure relevant intermediates, and their relevance to the observed increase in EPO levels requires dedicated mechanistic studies. An additional, indirect pathway may be anti‐inflammatory. Pro‐inflammatory cytokines such as interleukin (IL)‐1β and tumor necrosis factor‐α suppress EPO production (Cluzeau et al., 2017), and BHB inhibits the NOD‐like receptor pyrin‐domain containing 3 inflammasome (NLRP3), which suppresses caspase‐1 activation and thereby reduces IL‐1β and IL‐18 secretion in human monocytes (Youm et al., 2015). Relief of low‐grade inflammatory suppression of EPO is therefore plausible. However, this interpretation is tempered by inconsistent human data: although in vitro and animal studies support NLRP3 inhibition by BHB, acute exogenous ketone failed to attenuate lipopolysaccharide‐stimulated caspase‐1 activation in humans with obesity (Neudorf et al., 2020), and our participants were healthy with low baseline inflammatory burden. As cytokines were not measured, this pathway remains speculative.

### 4.2 Erythropoietic responses

KE did not alter hemoglobin levels in either sex. While RBC and MCV remained stable, MCHC was modestly but consistently lower with KE than with PBO at 2 weeks in both sexes. This pattern, a small reduction in MCHC without a change in cell volume, may reflect an increased proportion of newly produced, less haemoglobinised erythrocytes in circulation, suggesting that the observed EPO elevation may have begun translating into bone marrow output, although net hemoglobin and red cell mass remained unchanged. Reticulocyte counts, a direct marker of erythropoietic output, were not included in this study, so the MCHC pattern is consistent with, but does not directly demonstrate, increased newly produced erythrocytes. In females, hematocrit showed only a non‐significant trend toward increase with KE. To assess whether this reflected hemodilution or haemoconcentration, we examined surrogate markers of volume status (Tables S1 and S2). Creatinine and eGFR were stable, arguing against plasma volume contraction. Plasma sodium was an average of 2.7 mmol/L lower in females receiving KE, consistent with mild natriuresis from KE ingestion (Lyksholm et al., 2026), indicating hemodilution rather than haemoconcentration. Surprisingly, albumin was elevated in females receiving KE but not males, potentially reflecting an anti‐catabolic effect of β‐hydroxybutyrate rather than volume‐mediated changes (Thomsen et al., 2018). Thus, while volume‐mediated changes appear unlikely, the biological mechanism underlying the observed changes in MCHC and hematocrit trend in females remains unclear and warrants investigation in larger studies with reticulocyte enumeration.

### 4.3 Iron metabolism

The absence of detectable change in hemoglobin and red cell mass, despite signs of early erythropoietic stimulation, suggests that ketosis may facilitate iron utilization without depleting stores over this timeframe. Notably, the clinically relevant increase in ferritin levels observed in females aligns with their higher prevalence of iron deficiency (Sim et al., 2019; Weyand et al., 2023). This contrasts with some prior work on β‐hydroxybutyrate supplementation that did not alter iron markers in prediabetic individuals (Kimita et al., 2021), underscoring the importance of differentiating exogenous ketosis from endogenous metabolic shifts (e.g., fasting or ketogenic diets) which have shown inconsistent effects on iron regulation (Borkowska et al., 2021; Dyńka et al., 2023). An increased insulin response with oral intake of both ketone monoester (Vestergaard et al., 2021) and BHB salt (Rittig et al., 2020), though not measured here, may have contributed to the higher ferritin and plasma iron levels through downregulation of hepcidin and stimulatory effects on ferritin synthesis in macrophages (Fernández‐Real et al., 2015). Whether a sustained intermittent exogenous ketosis regimen can induce meaningful hematological changes in populations with high erythropoietic sensitivity, such as endurance athletes or those with relative iron deficiency, requires adequately powered research.

### 4.4 Sex hormones

The testosterone response in female participants contrasts with prior studies in women with polycystic ovary syndrome and obese populations, where ketosis decreased androgens (Rittig et al., 2025; Svart et al., 2024), likely reflecting population differences rather than a direct effect of ketosis. The inverse associations between EPO and both oestradiol and testosterone at baseline in females are biologically plausible: sex hormones modulate erythropoiesis, with estrogens generally inhibiting it (Murphy, 2014). The inverse EPO–sex hormone associations likely reflect estrogen‐mediated inhibition of erythropoiesis; oestradiol and testosterone co‐vary across the menstrual cycle in women but change independently in males, whose testosterone concentrations were approximately 25‐fold higher. Menstrual phase was neither recorded nor controlled during randomization, limiting interpretation of these baseline associations. That neither association persisted at day 14 suggests baseline hormonal heterogeneity rather than ketosis‐driven EPO suppression. Given the small sample size, these findings are exploratory and warrant investigation in adequately powered studies.

### 4.5 Strength and limitations

This study has several strengths. Participants were randomly assigned to BHB KE or PBO drinks matched for taste, volume, and viscosity, and the inclusion of healthy adults of both sexes across a reasonable age range supported generalisability. Compliance was monitored quantitatively after 1 week using a validated HPLC‐MS/MS method (Sørensen et al., 2013) and continuous ketone monitoring further confirmed intermittent ketosis in a subset of participants (Kjær et al., 2026). Since point‐of‐care devices are validated against HPLC‐MS/MS (Bjerg et al., 2025), our results remain directly comparable with studies employing alternative methods. A particular methodological strength was that the allocation sequence was generated only after enrolment had been completed, precluding any influence of anticipated allocation on participant selection.

This study has several limitations. Part 1 lacked a control group, and although a carry‐over effect into Part 2 is unlikely given the short duration of supraphysiological ketosis (5–7 h), it cannot be formally excluded. Part 1 was not designed to investigate EPO and did not contribute to the sample size calculation; it served to characterize the acute response in line with prior work (Lauritsen et al., 2018). Investigators were not blinded to allocation, which is inherent to supplementation trials of this design; however, outcome assessments were laboratory‐based and objective, thus mitigating bias.

The dosing schedule fixed 50% of each daily dose at bedtime, standardizing the principal overnight ketotic window, but allowed the remaining half to be taken in one or two daytime installments according to participant preference. This introduced inter‐individual variability in the precise timing of daytime ketone exposure, which we did not record in detail. Continuous ketone monitoring in a subset nonetheless confirmed that an intermittent ketosis pattern was achieved (Kjær et al., 2026). Dietary intake and physical activity were not prescribed, recorded, or controlled during the two‐week intervention; participants were asked to maintain their lifestyle, but this was not verified. Both behaviors can influence EPO, iron status, and hematological indices, so unrecorded between‐group differences cannot be excluded as contributors to observed effects. Randomization should distribute these influences across arms, but with this sample size that cannot be assured.

The small sample size limits statistical power, especially for sex‐stratified analyses, which are exploratory. Menstrual cycle phase heterogeneity among female participants may have affected hormonal measurements and EPO regulation, necessitating a cautious interpretation of sex‐specific endocrine findings. Recent hypoxia exposure (e.g., air travel, high‐altitude stays) was not an exclusion criterion; none were reported during the intervention, but prior exposures could not be excluded. The two‐week timeframe precluded the detection of hemoglobin or RBC mass changes, given the erythrocyte lifespan and the lag between EPO signaling and circulating RBC production. Reticulocyte count, which would have provided direct evidence of erythropoietic stimulation, requires fresh blood and was not incorporated into the study protocol. Generalisability is limited by the healthy study population, and results may not apply to clinical populations or those with altered metabolic profiles.

## 5 CONCLUSION

Two weeks of intermittent exogenous ketosis increased EPO concentrations (more marked in females) and reduced MCHC uniformly, consistent with early erythropoietic stimulation, whereas ferritin levels increased in females. However, hemoglobin and red cell mass were unchanged, suggesting that the timeframe was insufficient to detect cumulative erythropoietic output.

## AUTHOR CONTRIBUTIONS

**Henrik Holm Thomsen:** Conceptualization; data curation; formal analysis; funding acquisition; investigation; methodology; project administration; resources; validation; visualization. **Birgitte Sandfeld‐Paulsen:** Conceptualization; data curation; funding acquisition; investigation; methodology; project administration; resources. **Lise Nørkjær Bjerg:** Conceptualization; data curation; funding acquisition; investigation; methodology; project administration; resources. **Jesper Nørgaard Bech:** Conceptualization; data curation; formal analysis; investigation; resources.

## FUNDING INFORMATION

The investigation was funded through undisclosed research resources within the Medical Diagnostics Center, Regional Hospital Central Jutland, Viborg, and research grants from the Research Fund of Regional Hospital Central Jutland and the Clinical Academic Group for Multimorbidity at Regional Hospital Central Jutland. Funders were not involved in any part of the design, conduct, analysis, and reporting of the trial.

## CONFLICT OF INTEREST STATEMENT

The authors have no relevant financial or non‐financial interests to disclose.

## ETHICS STATEMENT

The study was conducted in accordance with the guidelines of the Declaration of Helsinki and approved by the Central Denmark Region Committee on Biomedical Research Ethics (1‐10‐72‐221‐22).

## CONSENT

Oral and written informed consent was obtained from all participants included in the study.

## Supporting information


**Figure S1:** Study flowchart. Two‐part study: Part 1, acute ketosis from ingestion of beta‐hydroxybutyrate (BHB) ketone monoester (KE) drink, followed by Part 2, randomized assignment to either a regimen of intermittent exogenous ketosis using a BHB KE drink taken twice daily or a placebo (PBO) vehicle matched in taste, volume, and viscosity, also consumed twice daily (*N* = 16).
**Figure S2:** CONSORT flow diagram.
**Figure S3:** Erythropoietin and testosterone: sex‐specific correlations stratified by study phase. Linear regression with cluster‐robust standard errors. Panels show baseline (a, c) and day 14 (b, d) data for females (a, b, *n* = 7) and males (c, d, *n* = 9). Fitted lines with 95% confidence intervals. In females, a significant inverse EPO–testosterone association at baseline (*p* = 0.035) attenuated by day 14 (*p* = 0.25). Males showed no significant association at either timepoint.
**Figure S4:** Erythropoietin and oestradiol: sex‐specific correlations stratified by study phase. Linear regression with cluster‐robust standard errors. Panels show baseline (a, c) and day 14 (b, d) data for females (a, b, *n* = 7) and males (c, d, *n* = 9). Fitted lines with 95% confidence intervals. In females, a trend toward inverse EPO–oestradiol association at baseline (*p* = 0.08) did not reach significance and weakened further by day 14 (*p* = 0.47). Males showed no significant association at either timepoint.
**Table S1:** Volume status markers at baseline and 180 min (Part 1). Data are means or ‡geometric means (95% CI) from the linear mixed‐effects model. eGFR, estimated glomerular filtration rate.
**Table S2:** Volume status markers at day 14 (KE vs. PBO), stratified by sex. Data are means or ‡geometric means (95% CI) from the linear mixed‐effects model (KE, *n* = 8; PBO, *n* = 8). eGFR, estimated glomerular filtration rate; KE, ketone monoester; PBO, placebo.

## Data Availability

The data supporting this study will be made available upon reasonable request.
