# Supplementary material for: Intermittent exogenous ketosis increases erythropoietin in healthy adults: A randomized, placebo‐controlled study with preliminary sex‐specific observations
Source: Physiol Rep. 2026 Aug 3;14(15):e71041. doi: 10.14814/phy2.71041 (PMC13433829; doi:10.14814/phy2.71041)
Supplement: Supplementary file 1 [file PHY2-14-e71041-s001.docx]

SUPPLEMENTARY FIGURES AND TABLES

# FIGURE S1

**Figure S1** Study flowchart. Two-part study: Part 1, acute ketosis from ingestion of beta-hydroxybutyrate (BHB) ketone monoester (KE) drink, followed by Part 2, randomised assignment to either a regimen of intermittent exogenous ketosis using a BHB KE drink taken twice daily or a placebo (PBO) vehicle matched in taste, volume, and viscosity, also consumed twice daily (N=16).

# FIGURE S2

**Figure S2.** CONSORT Flow Diagram

# FIGURE S3

**Figure S3.** Erythropoietin and testosterone: sex-specific correlations stratified by study phase. Linear regression with cluster-robust standard errors. Panels show baseline (A, C) and day 14 (B, D) data for females (A, B, n=7) and males (C, D, n=9). Fitted lines with 95% confidence intervals. In females, a significant inverse EPO–testosterone association at baseline (P = 0.035) attenuated by day 14 (P = 0.25). Males showed no significant association at either timepoint.

# FIGURE S4

**Figure S4**. Erythropoietin and oestradiol: sex-specific correlations stratified by study phase. Linear regression with cluster-robust standard errors. Panels show baseline (A, C) and day 14 (B, D) data for females (A, B, n=7) and males (C, D, n=9). Fitted lines with 95% confidence intervals. In females, a trend toward inverse EPO–oestradiol association at baseline (P = 0.08) did not reach significance and weakened further by day 14 (P = 0.47). Males showed no significant association at either timepoint.

# TABLE S1

|  | Baseline | 180 min. | P-value |
| --- | --- | --- | --- |
| Sodium, mmol/L | 141 (140, 142) | 141 (140, 141) | 0.47 |
| Creatinine, µmol/L | 79 (73, 85) | 76 (71, 81) | 0.008 |
| eGFR, mL/min/1.73 m²‡ | 94 (85, 102) | 98 (90, 105) | 0.006 |
| Albumin, g/L | 41 (40, 42) | 41 (39, 42) | 0.26 |

**Table S1.** Volume status markers at baseline and 180 min. (Part 1)**.** Data are means or ‡geometric means (95% CI) from the linear mixed-effects model. eGFR, estimated glomerular filtration rate

# TABLE S2

|  | KE | PBO | P-value |
| --- | --- | --- | --- |
| Sodium, mmol/L | 139 (139, 140) | 141 (140, 142) | 0.002 |
| *Males* | 140 (139, 141) | 141 (140, 142) | 0.44 |
| *Females* | 138 (137, 140) | 141 (140, 142) | 0.001 |
| Creatinine, µmol/L | 77 (72, 82) | 77 (72, 82) | 0.96 |
| *Males* | 85 (78, 91) | 85 (78, 91) | 0.96 |
| *Females* | 66 (59, 74) | 67 (60, 74) | 0.91 |
| eGFR, mL/min/1.73 m²‡ | 96 (89, 104) | 96 (89, 103) | 0.96 |
| *Males* | 97 (87, 107) | 96 (86, 106) | 0.94 |
| *Females* | 95 (87, 107) | 95 (84, 106) | 0.99 |
| Albumin, g/L | 44 (43, 45) | 43 (42, 44) | 0.06 |
| *Males* | 44 (43, 46) | 45 (43, 46) | 0.45 |
| *Females* | 43 (43, 46) | 40 (38, 41) | 0.002 |

**Table S2.** Volume status markers at day 14 (KE vs PBO), stratified by sex. Data are means or ‡geometric means (95% CI) from the linear mixed-effects model (KE, n = 8; PBO, n = 8). eGFR, estimated glomerular filtration rate; KE, ketone monoester; PBO, placebo.
